# Validation of a new emotion regulation self-report questionnaire for children

**DOI:** 10.1186/s12888-022-04440-x

**Published:** 2022-12-22

**Authors:** Michaela Junghänel, Hildegard Wand, Christina Dose, Ann-Kathrin Thöne, Anne-Katrin Treier, Charlotte Hanisch, Anne Ritschel, Michael Kölch, Lena Lincke, Veit Roessner, Gregor Kohls, Ulrike Ravens-Sieberer, Anne Kaman, Tobias Banaschewski, Pascal-M. Aggensteiner, Anja Görtz-Dorten, Manfred Döpfner, on behalf of the ADOPT Consortium, Dorothee Bernheim, Stefanie Bienioschek, Maren Boecker, Daniel Brandeis, Nina Christmann, Andrea Daunke, Jörg M. Fegert, Franziska Frenk, Franziska Giller, Claudia Ginsberg, Carolina Goldbeck, Monja Groh, Martin Hellmich, Sarah Hohmann, Nathalie Holz, Christine Igel, Anna Kaiser, Betül Katmer-Amet, Josepha Katzmann, Sabina Millenet, Kristina Mücke, Anne Schreiner, Jennifer Schroth, Anne Schüller, Marie-Therese Steiner, Marion Steiner, Anne Uhlmann, Matthias Winkler, Sara Zaplana

**Affiliations:** 1 School of Child and Adolescent Cognitive Behavior Therapy (AKiP), Faculty of Medicine and University Hospital Cologne, University of Cologne, Pohligstraße 9, 50969 Cologne, Germany; 2 Department of Special Education, Faculty of Human Sciences, University of Cologne, Cologne, Germany; 3 Department of Child and Adolescent Psychiatry/Psychotherapy, University of Ulm, Ulm, Germany; 4 Department of Child and Adolescent Psychiatry, Neurology, Psychosomatics, and Psychotherapy, University Medical Center Rostock, Rostock, Germany; 5 Department of Child and Adolescent Psychiatry, Psychotherapy and Psychosomatics, University Hospital Ruppin-Brandenburg, Neuruppin, Germany; 6 Department of Child and Adolescent Psychiatry and Psychotherapy, TU Dresden, Dresden, Germany; 7 Department of Child and Adolescent Psychiatry, Psychotherapy, and Psychosomatics, Research Unit Child Public Health, University Medical Center Hamburg-Eppendorf, Hamburg, Germany; 8 Department of Child and Adolescent Psychiatry and Psychotherapy, Central Institute of Mental Health, Medical Faculty Mannheim, University of Heidelberg, Mannheim, Germany; 9 Department of Child and Adolescent Psychiatry, Psychosomatics and Psychotherapy, Faculty of Medicine and University Hospital Cologne, University of Cologne, Cologne, Germany

**Keywords:** Emotion regulation, Affective dysregulation, Children, Adolescents, Assessment

## Abstract

**Objective:**

To examine and validate the self-report Questionnaire on the Regulation of Unpleasant Moods in Children (FRUST), which is a modified and shortened version of the Questionnaire for the Assessment of Emotion Regulation in Children and Adolescents (FEEL-KJ).

**Methods:**

The data comprised child and parent ratings of a community-screened sample with differing levels of affective dysregulation (AD) (*N* = 391, age: *M* = 10.64, *SD* = 1.33, 56% male). We conducted latent factor analyses to establish a factor structure. Subsequently, we assessed measurement invariance (MI) regarding age, gender, and AD level and evaluated the internal consistencies of the scales. Finally, we examined the convergent and divergent validity of the instrument by calculating differential correlations between the emotion regulation strategy (ERS) scales and self- and parent-report measures of psychopathology.

**Results:**

A four-factor model, with one factor representing *Dysfunctional Strategies* and the three factors *Distraction*, *Problem-Solving* and *Social Support* representing functional strategies provided the best fit to our data and was straightforward to interpret. We found strong MI for age and gender and weak MI for AD level. Differential correlations with child and parent ratings of measures of psychopathology supported the construct validity of the factors.

**Conclusions:**

We established a reliable and valid self-report measure for the assessment of ERS in children. Due to the reduced number of items and the inclusion of highly specific regulatory behaviors, the FRUST might be a valuable contribution to the assessment of ER strategies for diagnostic, therapeutic, and research purposes.

**Supplementary Information:**

The online version contains supplementary material available at 10.1186/s12888-022-04440-x.

## Background

Given that childhood and adolescence is accompanied by many challenges that elicit intense emotions [1], there has been a growing interest in understanding emotion regulation strategies (ERS) and their development in childhood and adolescence [2]. One way to classify ERS is to differentiate between maladaptive or dysfunctional and adaptive or functional strategies based on their immediate effects on affect, cognition, and behavior [3]. An adequate use of functional ERS allows “for monitoring, evaluating, and modifying emotional reactions, especially their intensive and temporal features” ([4], pp. 27–28). By comparison, the limited use of functional ERS and the use of dysfunctional ERS have been linked to psychopathology (e.g. [3]). For example, the dysfunctional ERS *rumination* is common in depressive disorders (e.g. [5]), whereas in most anxiety disorders, the object of fear is *avoided* in order to prevent negative emotions (e.g. [6]). *Avoidance* has also been shown to be associated with higher levels of attention-deficit/hyperactivity disorder (ADHD [7];). Moreover, studies have demonstrated that the limited use of the functional ERS *problem-solving, acceptance*, and *reappraisal* is associated with internalizing and externalizing symptoms in general [8, 9]. Specifically, Braet and colleagues [9] found that internalizing symptomatology in school-aged children was negatively associated with the functional ERS *problem-oriented action, distraction, acceptance, forget*, and *revaluation* and that externalizing symptomatology was negatively associated with the functional ERS *problem-oriented action* and *acceptance*.

Numerous studies have found associations of ERS with gender and age, albeit with inconsistent results. For instance, some studies found that ERS steadily increase with age (e.g. [2, 10, 11]), which is consistent with the cognitive maturation model [12]. In addition to this general increase in functional ERS, it was found that the kinds of strategies applied also change significantly during childhood and adolescence [2, 11, 13]. By contrast, other studies (e.g. [11, 13]) reported evidence supporting the maladaptive shift model [12], which assumes a decrease in functional ERS and an increase in dysfunctional ERS in (early) adolescence. Taken together, these results suggest that efficacy and flexibility in the use of ERS increase with age, with an interruption of a few years during adolescence, in which emotion regulation (ER) temporarily deteriorates [12, 13]. Regarding the effect of gender on ER, numerous studies suggest that girls show fewer functional and more dysfunctional ERS than do boys [12, 13]. For example, in a sample of 8 to 14-year-olds, girls showed significantly decreased functional ERS and significantly increased dysfunctional ERS compared to boys, and this effect was particularly pronounced in females in grades five and six (corresponding to age 10–12 [13];). This finding may potentially be attributable to higher stress levels [13] and a more intense experience of emotions [14] in girls than in boys, or different socialization processes of girls and boys [15]. Other findings offer a more nuanced view on the association between gender and ER, suggesting that boys and girls apply different ERS. In particular, functional *social support seeking* and dysfunctional *rumination* seem to be more prominent in girls [11, 13], whereas boys have been found to apply passivity, avoidance and suppression more frequently [11].

The term emotion dysregulation is often used synonymously with the terms affective dysregulation (AD) and irritability, which all refer to a highly similar and strongly overlapping construct [16–18]. However, as opposed to irritability, the concepts of emotion dysregulation and AD generally include an irritable *and* an impulsive component [16, 19]. Moreover, it is consensus that emotion (dys-)regulation primarily describes the *process of coping* with all kinds of emotional challenges whereas AD describes an *emotional state* that may result from emotion dysregulation [19–21]. Conversely, the expression of negative emotions may also be one of several dysfunctional coping processes [22]. This being said, it is important to emphasize that the expression of negative emotions per se is not negative as an adequate expression of negative emotions can serve important social functions (e.g. the expression of sadness can lead other people to comfort us; the expression of anger can help set necessary boundaries in social contexts). Importantly, emotion dysregulation is not synonymous with any single mental disorder defined in the 5th edition of the Diagnostic and Statistical Manual of Mental Disorders (DSM-5 [23];) but has been shown to be transdiagnostic and consequently associated with various externalizing and internalizing disorders [2]. At its most extreme it may reflect the new DSM-5 diagnosis of “disruptive mood dysregulation disorder” (DMDD [24];). DMDD is characterized by severe, recurrent temper outbursts and chronic irritability or angry mood. By comparison, the International Statistical Classification of Diseases and Related Health Problems (ICD-11 [25];) takes a different approach to describing a similar pathology by adding a specifier for chronic irritability to the diagnosis of oppositional defiant behavior (ODD), thus reflecting the difficulties to disentangle irritability and ODD symptomatology [16, 26].

In sum, difficulties in ER are associated with a variety of externalizing and internalizing psychiatric disorders. Moreover, ERS are especially important for the examination of the newly introduced diagnostic entities of DMDD (DSM-5 [23];) or ODD with chronic irritability (ICD-11 [25];), both introduced to better capture AD symptomatology. Furthermore, open questions remain with regard to associations of ER with gender and age. Therefore, a valid and reliable assessment of a range of functional and dysfunctional ERS for diagnostic, therapeutic, and research purposes seems crucial.

There are a number of internationally used questionnaires assessing ERS in children and adolescents. A meta-analysis by Compas and colleagues [2] found 87 distinct measures of ERS, with the most commonly used being the parent-reported Emotion Regulation Checklist (ERC [27];), which assesses the two dimensions of Negativity and ER with a total of 24 items, and the 36-item self-report Cognitive Emotion Regulation Questionnaire (CERQ [28];), which assesses nine cognitive coping strategies. Other frequently used questionnaires include the self-report Emotion Regulation Questionnaire for Children and Adolescents (ERQ-CA [29];), which is an adapted version of the Emotion Regulation Questionnaire for adults [30], and the Children’s Response Style Questionnaire (CRSQ [31, 32];). Both of these measures assess a limited number of strategies (ERQ-CA: ten items, Cognitive Reappraisal and Expressive Suppression; CRSQ: 25 items, Rumination, Distraction, Problem-Solving). The main advantages of all of the aforementioned questionnaires lie in their reasonable number of items (10–36) as well as their sound psychometric quality. However, they all come with problems, such as the lack of a self-report measure (ERC) or the limited number of strategies assessed (CERQ, ERQ-CA, CRSQ).

The self-report Questionnaire for the Assessment of Emotion Regulation in Children and Adolescents (FEEL-KJ [33, 34];), for children between the ages of 10;0 and 19;11 years, is a well-established and frequently used instrument to assess the application of ERS in children and adolescents [35–37]. With a total of (15x2x3=) 90 items, the FEEL-KJ assesses 15 different ERS, with each strategy assessed independently with two items for the three emotions anger, sadness, and anxiety. Accordingly, the FEEL-KJ allows for an emotion-specific evaluation as well as a comprehensive evaluation of ERS across the three emotions. As reported by Grob and Smolenski [33], the different strategies can be combined into the higher-order scales *adaptive emotion regulation strategies* and *maladaptive emotion regulation strategies*. However, Cracco and colleagues [34] suggested a more complex factor structure, subdividing the maladaptive factor into the three lower-level factors *avoidance*, *dysfunctional thoughts*, and *aggression*. The FEEL-KJ overcomes the disadvantage of several ER measures that assess only a limited number of strategies, although due to the detailed assessment of various ERS, each rated for the three different emotions anger, sadness, and anxiety, the 90-item FEEL-KJ is quite lengthy and repetitive. The distinction between the ERS in response to the three emotions seems particularly questionable as the internal consistency and the test-retest reliability for most primary scales for the emotion-specific evaluation are weak [38]. Furthermore, three of the strategies (expression, social support, control of emotions) could not be unequivocally assigned to either of the secondary scales, as associations with psychological well-being were not unambiguously positive or negative [38].

## Aim of study

In light of the shortcomings of the FEEL-KJ, especially its length and repetitive elements, and to be able to monitor therapeutic processes, we revised and adapted this questionnaire, resulting in the newly developed 48-item self-report “Questionnaire on the Regulation of Unpleasant Moods in Children” (German: “Fragebogen zur Regulation unangenehmer Stimmungen” [FRUST]) by *[masked for blind review]*, unpublished manuscript). The aim of the present study was to examine the factor structure of the FRUST in a sample consisting of children with either no or pronounced AD symptomatology. Furthermore, we aimed to assess whether the observed factor structure was invariant to gender, age, and AD level. In addition, we examined the internal consistencies of the resulting scales as well as their correlations with one another. To examine the convergent and divergent validity of the FRUST, we considered the associations of its scales with measures of AD, symptoms of ADHD, symptoms of ODD, anxious/depressed symptoms, and aggressive symptoms. Finally, we analyzed the associations of the FRUST scales with gender and age of the participants.

## Methods

### Participants and procedure

Data collection took place within the ongoing multicenter research project *[masked for blind review]*, which encompasses *[masked for blind review]*. The multicenter research project aims to optimize the diagnostic investigation, prevention, and treatment of AD and includes several measurement time points. For the present study, we used the baseline data (T1) of a community-screened sample, which was recruited through the local residents’ registration office and was one of various different samples considered in the research project. The data were collected between August 2018 and September 2019 (for further information see the study protocol; *[masked for blind review]*). The sample includes *n* = 391 children aged 8 to 12 years (*M* = 10.64, *SD* = 1.33; 56% males). The mothers of 342 (88%) and the fathers of 46 (12%) of these children completed the parent questionnaires used for the current analyses. Main inclusion criteria were the age of the child (8;0–12;11 at T1), child living with at least one biological or adoptive parent, status of AD symptomatology of the child, as well as willingness and ability to participate in the study. Prior to the baseline measurement, participants were assigned to an AD or a NoAD group, based on a predefined cut-off score on a parent screening questionnaire (*[masked for blind review;* for further details regarding the screening procedure see *[masked for blind review]).* Children with AD symptom scores in the top 10% of the sample were allocated to the AD group and children with scores in the bottom 10% were allocated to the NoAD group. All families from the AD group and a random sample from the NoAD group were asked to participate further in the study. Participating families underwent clinical child and parent interviews *[masked for blind review]*, with the latter being used to confirm the child’s AD status. As shown in Table 1, a total of 244 (62%) study participants were assigned to the AD group. Clinical interviews with the parents (*[masked for blind review]* by *[masked for blind review],* in press) were conducted to examine whether the children and adolescents met DSM-5/ICD-10 criteria for a mental disorder. All diagnoses can be found in Table 1.

**Table 1:** Sample and descriptive statistics

*Sample Statistics*	
Total sample	*n* = 391
Age: mean (*SD*)	10.64 (1.33)
Male: *n* (%)	220 (56)
Group: *n* (%)	
AD	244 (62)
NoAD	147 (38)
Diagnoses: *n* (%)	
DMDD^a^	41 (11)
ODD^a^	93 (24)
ADHD	62 (16)
ADHD, combined type	19 (5)
ADHD, predominantly inattentive type	30 (8)
ADHD, predominantly hyperactive-impulsive type	13 (3)
CD	6 (2)
MD	5 (1)
No Diagnosis	262 (67)

*SD* Standard deviation, *M* Mean, *AD* Affective dysregulation, *ADHD* Attention-deficit/hyperactivity disorder, *DMDD* Disruptive mood dysregulation disorder, *ODD* oppositional defiant disorder, *AD* Affective dysregulation, *CD* Conduct disorder, *MD* Major depressive episode

^a^Although not possible in the DSM-5 due to hierarchical rules, simultaneous DMDD and ODD diagnoses are reported here if all diagnostic criteria were met, due to interest in the diagnostic overlap

### Measures

#### FRUST

The Questionnaire on the Regulation of Unpleasant Moods in Children (FRUST; *[masked for blind review],* unpublished manuscript) is an adaptation of the FEEL-KJ questionnaire [33], which assesses ERS in children and adolescents. The originally developed version of the FRUST comprises 48 items and is thus shorter than the FEEL-KJ. While the FEEL-KJ assesses the same ERS in response to anger, sadness, and anxiety separately, the FRUST combines the response to these three emotions and assesses the strategies for the regulation of “unpleasant emotions” in general. In addition to the 30 items originating from the FEEL-KJ, the FRUST questionnaire includes 18 new items, which refer to interventions used in the scope of the Treatment Program for Children with Aggressive Behavior (THAV; German: “Therapieprogramm für Kinder mit aggressivem Verhalten” [39];). These items target very specific behaviors taught in the THAV, that can be applied as ERS when feeling bad (e.g. “*When I feel bad, I count to ten*” or “*When I feel bad, I do a relaxation exercise*”), as the THAV program has been shown to reduce aggressive behaviors and increase prosocial behavior in children with peer-related aggressive behaviors [40, 41], which is closely associated to AD symptomatology. The items are rated on a five-point Likert-type scale ranging from 0 (almost never) to 4 (almost always).

#### DADYS-p/DADYS-c

Child AD symptoms were assessed using the self- and parent report forms of the Diagnostic System for Affective Dysregulation (DADYS; German: “Diagnostikum für Affektive Dysregulation”; unpublished manuscript [42];). The self-report form (DADYS-C) comprises 28 items and the parent report form (DADYS-P) comprises 38 items. The DADYS items originate from several existing questionnaires, that is the Emotion Regulation Checklist [27], the German Symptom Checklist for Disruptive Behavior Disorder (FBB/SBB-SSV; German: “Fremd-/Selbstbeurteilungsbogen für Störungen des Sozialverhaltens” [43];), and the Affective Reactivity Index [44]. These questionnaires assess irritability/anger and/or affective dysregulation/emotion regulation, leading to a rather broad conceptualization of AD in the present study. All items of the DADYS-C and DADYS-P are rated on a four-point Likert-type scale ranging from 0 (not at all/never) to 3 (very much/always), with higher scores indicating higher symptom severity. A total score was computed by summing up all item scores and dividing this sum by the number of items, resulting in a total score ranging between 0 and 4. The internal consistencies of the total scale scores in the present sample were good, with α = .94 (*M* = .89, *SD* = .50) for the DADYS-C and α = .96 (*M* = .89, *SD* = .56) for the DADYS-P.

#### CBCL/6-18R

The German version of the Child Behavior Checklist for Ages 6–18 (CBCL/6-18R [45];), originally developed by Achenbach [46], comprises 120 items assessing a range of behavioral and emotional problems in children and adolescents. Parents rate the items on a three-point Likert scale ranging from 0 (not true) to 2 (very true). The items can be aggregated to eight syndrome scales (Anxious/Depressed, Withdrawn/Depressed, Somatic Complaints, Social Problems, Thought Problems, Attention Problems, Rule-Breaking Behavior, Aggressive Behavior) and three broadband scales (Externalizing Problems, Internalizing Problems, Total Problems). In this study, we assessed scales of the CBCL dysregulation profile [47, 48], which includes the scales *Anxious/Depressed* (part of the Internalizing Problems scale), *Attention Problems* (part of the total problems scale) and *Aggressive Behavior* (part of the Externalizing Problems scale). The scale scores were computed by averaging the associated item scores. The syndrome scale scores of these three scales as well as the broadband scales have shown good psychometric properties [45]. In the present sample, all assessed syndrome scales demonstrated good internal consistencies, with α = .83 for the *Anxious/Depressed* scale (*M* = .31, *SD* = 0.31), α = .87 for the *Attention Problems* scale (*M* = .44, *SD* = 0.43), and α = .93 (*M* = .45, *SD* = 0.40) for the *Aggressive Behavior* scale.

#### SBB−/FBB-ADHS

The children completed the self-report (SBB-ADHS) and the mothers or fathers the parent report (FBB-ADHS) form of the German Symptom Checklist for Attention-Deficit/Hyperactivity Disorder (German: “Selbst-/Fremdbeurteilungsbogen für Aufmerksamkeitsdefizit-/Hyperaktivitätsstörungen”) from the German Diagnostic System for Mental Disorders in Children and Adolescents based on the ICD-10 and DMS-5 (DISYPS-III [43];). The two forms comprise 20 items each, which are rated on a four-point Likert scale ranging from 0 (not at all) to 3 (very much). The two subscales *Inattention* (nine items) and *Hyperactivity/Impulsivity* (eleven items) as well as a total score can be computed by averaging the associated item scores. The FBB-ADHS has demonstrated a stable factor structure. Moreover, previous research yielded good reliability and validity of the scale scores [49]. Internal consistencies in the present sample were good, with α = .91 (*M* = .60, *SD* = 0.47) for the SBB-ADHS total score and α = .95 (*M* = .73, *SD* = 0.65) for the FBB-ADHS total score.

#### SBB−/FBB-SSV

The children completed the self-report (SBB-SSV) and the mothers or fathers the parent report (FBB-SSV) form of the German Symptom Checklist for Disruptive Behavior Disorders (German: “Selbst-/Fremdbeurteilungsbogen für Störungen des Sozialverhaltens”) from the DISYPS-III [43]. The SBB−/FBB-SSV comprises a total of 37 items, of which eight items assess symptoms of oppositional defiant disorder (ODD), three items assess DMDD, 15 items assess symptoms of conduct disorder (CD), and 11 items assess callous-unemotional (CU) symptoms. All items are based on DSM-5 and ICD-10 symptom criteria. Following the questionnaire’s instruction to assess CD and CU symptoms only in children aged 11 years or older and as the DMDD items were included in the DADYS questionnaires assessing AD, we only used the items assessing ODD for the present study. Three of the eight items assessing ODD were already included in the DADYS questionnaire and were therefore excluded, resulting in five items assessing the defiant dimension of ODD (ODD^D^) in the current sample. All items are rated on a four-point Likert scale ranging from 0 (not at all) to 3 (very much), and a scale score can be derived by averaging the item scores. A stable factor structure of the FBB-SSV as well as good reliability of the scale scores and diagnostic accuracy have been demonstrated [50]. Internal consistencies in the present sample were good, with α = .85 (*M* = .68, *SD* = 0.53) for the SBB-ODD^D^ scale and α = .91 (*M* = .89, *SD* = 0.73) for the FBB-ODD^D^ scale.

### Statistical analyses and analysis plan

To examine the factor structure of the FRUST, we followed both an exploratory and a subsequent confirmatory factor analytic approach. The respective analyses were performed using Mplus version 8.4 [51]. For both analyses, we used the weighted least square mean and variance adjusted (WLSMV) estimator (delta parameterization), which is recommended for ordinal data [52]. The percentage of missing data per item was ≤0.3% for all items; missing data were handled using pairwise deletion. Covariance coverage was ≥ .995 for all items. We based our evaluation of model fit on the following frequently used global model fit indices: the comparative fit index (CFI), the Tucker-Lewis index (TLI), the root mean square error of approximation (RMSEA), and the standardized root mean square residuals (SRMR). Good model fit was indicated by CFI and TLI values ≥ .95 [53] as well as RMSEA and SRMR values ≤ .05 [54, 55]. For model fit to be considered as adequate, RMSEA and SRMR values should be ≤ .08 and CFI and TLI values ≥ .90 [54–56].

We first applied an exploratory principal axis factor analysis (estimator: WLSMV; delta parameterization). We chose an exploratory approach as we had no clear hypothesis about the underlying factor structure. In this study, we were interested in extracting factors that distinguish between rather functional versus rather dysfunctional ERS. As we did not expect all of the variance to be explained by the extracted factors and assumed correlations between the extracted factors, we used an oblique GEOMIN rotation. To determine a suitable number of factors to extract, we conducted a parallel analysis in Mplus, which is based on eigenvalues from the observed correlation matrix and compares these with eigenvalues of random variables. Factors that have larger eigenvalues than the random variables are chosen for further factor analytic examination [57]. We excluded items from further analysis if they showed substantial cross-loadings (> .30) on factors that were not compatible in terms of content (i.e. positive loadings on a factor otherwise defined through items describing dysfunctional ERS *and* a factor otherwise defined through items describing functional ERS) or if they only demonstrated factor loadings < .30, which is in accordance with Kline [58]. If substantial loadings (> .30) emerged on two factors that did not directly oppose each other (e.g., two factors defined through functional ERS), the item was assigned to one of the factors based on theoretical considerations.

Subsequently, we performed a confirmatory factor analysis (CFA) including all items that had not been excluded in the preceding exploratory analysis. In the CFA, we restricted items to load only onto a certain factor, thus eliminating cross-loadings. Items loading negatively on their respective factor were recoded such that higher item scores indicated a higher manifestation on this factor.

Following current recommendations [59–61], we assessed measurement invariance (MI) of our final model across different ages (8–10 years vs. 11–12 years), gender (male vs. female), and AD level (AD vs. NoAD). The different levels of invariance include an increasing number of restrictions. For configural invariance, the item-factor configuration is required to be equal across groups, that is, the same items have to belong to the same factor. Metric invariance can be assumed if the item loadings are additionally equivalent across groups. For scalar invariance, the item thresholds have to be equal across groups. The same goodness-of-fit indices as mentioned above (CFI, TLI, RMSEA, SRMR; theta parameterization) were used, and difference tests between the fit indices on different levels of invariance were computed to examine MI [60]. For the assessment of configural invariance, the same cut-off values as mentioned above can be applied. Additionally, a change in CFI of ≤ − .01 [62], a change in SRMR of ≤ + .03 for testing metric invariance and a change of CFI of ≤ − .01 and a change in SRMR of ≤ + .01 for testing scalar invariance [63], as well as equal or better fit of TLI and RMSEA [64] have been suggested to indicate invariance across groups under the imposed constraints.

To examine the internal consistency of the scales derived from the factor analyses, we computed Cronbach’s α. An α coefficient ≥ .70 was considered as adequate [65].

To examine the convergent and divergent validity of the corresponding scales derived from our latent factor analyses, we calculated Pearson correlation coefficients between the scales of the FRUST as well as between the FRUST scales and the CBCL/6-18R scales *Anxious/Depressed*, *Attention Problems*, *Aggressive Behavior*, the SBB−/FBB-ADHS, the SBB−/FBB-SSV ODD^D^ and the DADYS-C/−P. Moreover, correlations of the FRUST dimensions with age and gender were examined. With gender as a dichotomous variable, the point-biserial correlation was calculated. In accordance with Cohen [66] a correlation coefficient between .10 and .29 was considered small, a correlation coefficient between .30 and .49 as moderate, and a correlation coefficient > .50 as large. SPSS version 26 was used to calculate the internal consistency and the correlations.

## Results

### Factor structure and internal consistency of the FRUST

**Fig. 1:**
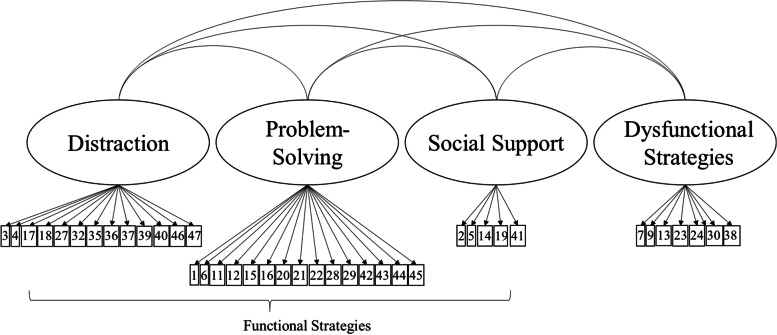
Final Four-Factor Structure of the FRUST. Correlated factors model with three functional factors (distraction, problem-solving, social support) and one dysfunctional factor after the exclusion of items 25 and 26 (due to low factor loadings). Item numbers are displayed in the boxes and residuals are not shown for clarity of presentation

The parallel analysis suggested a four-factor solution, which yielded a good model fit (CFI = .96, TLI = .95, RMSEA = .045 [90% CI: .042–.049], SRMR = .048) and was clearly interpretable (Table 2). Factors 1 to 3 comprised functional ERS, differentiated into *Distraction* (factor 1), *Problem-Solving* (factor 2) and *Social Support* (factor 3). Factor 4 encompassed *Dysfunctional ERS*. All four factors together explained 49% of the variance. Six items (items 8, 10, 31, 33, 34, 48) were excluded from further analyses due to substantial factor loadings on both a functional factor and the dysfunctional factor (see Table 2). In a next step, the model with four correlated factors derived from the EFA was tested using CFA, including the 42 remaining items. In this model, all cross-loadings were fixed to zero. All fit indices indicated an adequate fit of this more restricted model (CFI = .93, TLI = .93, RMSEA: .058 [90% CI: .055; .062], SRMR = .062). The factor loadings of items 25 (“*When I feel bad, I withdraw*”; factor *Dysfunctional Strategies*) and 26 (“*When I feel bad, I do not show my bad mood*”, factor *Problem-Solving*) were below .30 (.25 and .16, respectively). These items were therefore excluded, resulting in a final number of 40 items (Fig. 1). As item 5 (*“When I feel bad, I keep my feelings to myself”*) showed a negative factor loading on the functional *Social Support* factor, the scores for this item were recoded. This slightly adapted model was then tested in another CFA and yielded a somewhat improved fit (CFI = .94, TLI = .94, RMSEA: .058 [90% CI: .054–.062], SRMR = .059). The factor loadings for this final model can be found in Table S1. All items demonstrated a substantial loading on their respective factor (*a* ≥ .36). Moreover, all functional factors correlated positively with each other (*r* = .64 to .84, all *p* < .001) and negatively with the dysfunctional factor (*r* = −.50 to −.54, all *p* < .001).

**Table 2:** Item statistics and exploratory factor analysis of the FRUST – Four-Factor Solution

Items	*M* (*SD*)	Distraction	Problem-Solving	Social Support	Dysfunctional Strategies
When I feel bad, I …					
1 … try to change what made me feel bad	2.27 (1.22)	.08	.43*	.08	−.04
2 … tell someone about how I feel	2.09 (1.31)	−.04	.45*	.59*	−.08
3 … think about things that make me happy	2.45 (1.31)	.70*	.09	.02	−.06
4 … do something fun	2.82 (1.18)	.69*	.13	−.08*	−.05
5 … keep my feelings to myself	2.08 (1.28)	.09	−.02	−.64*	.17*
6 … make the best of it	2.22 (1.30)	.35*	.53*	−.22*	−.05
7 … do not want to see anyone	1.34 (1.31)	.01	−.26*	−.06	.59*
*8 … think that this is my own problem*	1.81 (1.22)	*−.05*	.41*	−.33*	.34*
9 … do not feel like doing anything	147 (1.30)	−.10	−.12	−.09	.59*
*10 … keep thinking about why I feel bad, without finding a solution*	1.25 (1.20)	*−.06*	.27*	*.02*	.37*
11 … think about what I could do	2.39 (1.28)	.35*	.47*	.12*	−.05
12 … tell myself that the problem is not that bad	1.75 (1.20)	.12	.54*	.02	.12*
13 … start a fight	0.80 (1.08)	.00	−.40*	−.01	.48*
14 … say that I am in a bad mood	1.84 (1.34)	−.06	.37*	.51*	.09
15 … try to forget what put me in a bad mood	2.14 (1.35)	.24*	.44*	.01	.01
16 … try to find the mistake in my own behavior	2.04 (1.28)	.06	.66*	−.06	.11*
17 … remember happy things	2.46 (1.38)	.70*	.16*	−.02	−.08
18 … try to make the best of a situation myself	2.26 (1.30)	.53*	.39*	−.05	−.14*
19 … go to someone who might be able to help me	2.08 (1.33)	.14*	.51*	.44*	.02
20 … think that it will pass	2.26 (1.28)	.10	.57*	−.03	.02
21 … accept what makes me feel bad	2.02 (1.22)	.07	.54*	−.01	.04
22 … show my bad mood without annoying others	185 (1.29)	−.03	.53*	.22*	.15*
23 … take my bad mood out on others	0.98 (1.16)	−.06	−.44*	−.01	.52*
24 … cannot get it out of my head	1.90 (1.33)	−.21*	.17*	−.10*	.54*
25 … withdraw	2.10 (1.30)	.00	.01	−.04	.43*
26 … do not show my bad mood	1.55 (1.20)	−.06	.42*	−.36*	.09
27 … do something I enjoy	2.51 (1.30)	.71*	.21*	−.04	−.11*
28 … think about a solution	2.33 (1.31)	.26*	.58*	.17*	−.09*
29 … tell myself that it is not important	1.38 (1.18)	−.10*	.60*	.06	.19*
30 … cannot do anything against my bad mood	1.34 (1.34)	−.30*	.01	−.12*	.47*
*31 … tell myself that I have to blow off steam to react cooler*	1.47 (1.32)	*.07*	.37*	*.01*	.38*
32 … do a relaxation exercise	0.87 (1.24)	.46*	.06	.34*	.18*
*33 … hit a pillow/go for a run*	1.36 (1.39)	*.06*	*.06*	.46*	.59*
*34 … squeeze something in my hand or make a fist in my pocket*	1.44 (1.34)	*.09*	*−.06*	.32*	.64*
35 … count to ten	0.46 (0.93)	.36*	−.01	.26*	.20*
36 … go to a nice place in my mind where I can relax	1.61 (1.46)	.65*	.06	.11*	.09
37 … listen to music/a story/watch a movie	2.09 (1.42)	.54*	.01	−.02	.19*
38 … hurt myself	0.55 (1.00)	−.14	−.11	.04	.58*
39 … play/paint/do handicrafts	1.77 (1.45)	.54*	.02	.16*	.07
40 … solve riddles/read	1.84 (1.45)	.60*	−.08	.20*	.08
41 … talk to someone about it	1.74 (1.36)	.02	.47*	.60*	−.09*
42 … try to stay cool and see the problem from another perspective	1.81 (1.30)	.13*	.66*	.01	−.01
43 … try to think smart instead of obsessing about it	2.10 (1.30)	.24*	.65*	−.03	−.13*
44 … leave the situation	2.25 (1.29)	.28*	.58*	.00	.02
45 … try to recognize the situation early that made me feel bad	2.08 (1.31)	.00	.76*	.07	.05
46 … eat something tasty	1.68 (1.38)	.57*	−.03	.01	.24*
47 … lie on my bed and dream about something nice/try to sleep	1.38 (1.33)	.53*	−.04	.08	.14*
*48 … do something on my computer/smartphone/tablet*	1.60 (1.48)	.14*	*.07*	−.36*	.19*

*M* Mean, *SD* Standard deviation

The extraction method was principal axis factoring with oblique (GEOMIN) rotation. Loadings marked in bold indicate the factor to which an item was assigned. Items that were excluded due to substantial factor loadings (> .30) on an functional and the dysfunctional factor are marked in italics

* *p* < .05

### Measurement invariance of the four-factor model of the FRUST across age, gender, and AD level

Regarding gender and age, the global model fit parameters as well as their change between the different levels of MI indicates that invariance can be assumed on a configural, metric, and scalar level. This suggests an equal factor organization, equivalent loading patterns, and equivalent item thresholds across age- and gender-related groups (see Table 3). For AD level, MI could be assumed on the configural and metric level, but not on the scalar level, as the change in CFI of −.26 was larger than the recommended cut-off of −.01. This suggests an equal factor organization and equivalent loading patterns, but differences in item thresholds (Table 3).

**Table 3:** Measurement Invariance for the Four-Factor Model of the FRUST

	female (*n* = 170) vs. male (*n* = 221)					AD (*n* = 244) vs. NoAD (*n* = 147)					8–10 years (*n* = 231) vs. 11–12 years (*n* = 160)				
Level of invariance	χ^2^ *(df)*	CFI	TLI	RMSEA [90% CI]	SRMR	χ^2^ (*df*)	CFI	TLI	RMSEA [90% CI]	SRMR	χ^2^ (*df*)	CFI	TLI	RMSEA [90% CI]	SRMR
configural	2358.247* (1468)	.943	.939	.056 [.052, .060]	.070	2239.890* (1318)	.932	.927	.060 [.056, .064]	.072	2339.512* (1468)	.945	.941	.055 [.051, .059]	.071
metric	2274.546* (1504)	.950 (∆ = .007)	.948	.051 [.047, .055]	.076 (∆ = .006)	2155.655* (1352)	.940 (∆ = .008)	.938	.055 [.051, .059]	.079 (∆ = .007)	2380.652* (1504)	.944 (∆ = −.001)	.942	.055 [.050, .059]	.081 (∆ = .010)
scalar	2481.617* (1660)	.947 (∆ = −.003)	.950	.050 [.046, .054]	.077 (∆ = .001)	2812.709* (1500)	.914 (∆ = −.026)	.920	.067 [.063, .071]	.095 (∆ = .016)	2571.871* (1660)	.942 (∆ = −.002)	.946	.053 [.049, .057]	.081 (∆ = .000)

*AD* Affective dysregulation, *CFI* Comparative fit index, *TLI* Tucker-Lewis index, *RMSEA* Root mean square error of approximation, *CI* confidence interval, *SRMR* Standardized root mean square residual

* *p* < .001

### Internal consistencies, scale Intercorrelations, and associations of the FRUST scales with psychopathology, gender, and age

Internal consistencies of the corresponding scales were adequate to good, with α = .78 for *Dysfunctional Strategies* (*M* = 1.19, *SD* = 0.80), α = .83 for *Social Support* (*M* = 1.94, *SD* = 1.01), α = .87 for *Distraction* (*M* = 1.86, *SD* = 0.83), and α = .91 for *Problem-Solvin*g (*M* = 2.05, *SD* = 0.84).

The positive scales of the FRUST demonstrated high positive correlations with each other (*r* = .51 to *r* = .71; all *p* < .01) and moderate negative correlations with the scale *Dysfunctional Strategies* (*r* = −.38 to *r* = −.40; all *p* < .01). The three functional ER scales showed significant (*p* < .01 or *p* < .05) small to large negative correlations with parent and child ratings of AD, ADHD, ODD^D^, and the three CBCL scales *Anxious/Depressed*, *Attention Problems*, and *Aggressive Behavior* (*r* = −.12 to *r* = −.57). The dysfunctional ER scale showed significant (all *p* < .01) small to large positive correlations with parent and child ratings of AD, ADHD, ODD^D^, and the three CBCL scales (*r* = .23 to *r* = .71). All FRUST scales demonstrated higher correlations with child ratings of AD, ADHD, and ODD^D^ symptomatology than with parent ratings of these variables on a descriptive level. There were no significant correlations between age and the FRUST scales. The functional strategies *Distraction* and *Social Support* showed small (*r* = .14 and *r* = .17, respectively) positive correlations with gender (Table 4).

**Table 4:** Scale intercorrelations, and associations of the FRUST scales with AD, ADHD, ODD^D^, the CBCL Scales and Age and Gender

	Distraction	Problem-Solving	Social Support	Dysfunctional
Distraction		.71**	.51**	−.38**
Problem-Solving			.61**	−.40**
Social Support				−.38**
DADYS-P	−.25**	−.30**	−.28**	.38**
DADYS-C	−.47**	−.57**	−.48**	.71**
FBB-ADHS	−.15**	−.18**	−.21**	.24**
SBB-ADHS	−.28**	−.34**	−.32**	.57**
FBB-ODD^D^	−.16**	−.24**	−.24**	.37**
SBB-ODD^D^	−.37**	−.47**	−.40**	.70**
CBCL-AD	−.12*	−.12*	−.12*	.23**
CBCL-AP	−.13*	−.18**	−.19**	.23**
CBCL-AB	−.18*	−.25**	−.26**	.36**
Age	.10	.01	.04	.01
Gender^a^	.14**	.00	.17**	.00

*DADYS* Diagnostic System for Affective Dysregulation (DADYS; German: “Diagnostikum für Affektive D**ys**regulation”), *P* Parent rating, *C* Child rating, *ADHD* Attention-deficit/hyperactivity disorder, *ODD*^*D*^ Oppositional defiant disorder – only defiant dimension, *CBCL* Child Behavior Checklist, *AD* Anxious/depressed, *AP* Attention problems, *AB* Aggressive behavior

^a^1 = male, 2 = female

* = *p* < .05

** = *p* < .0

## Discussion

The present study examined the factor structure, internal consistencies, and validity of the newly developed, self-report FRUST questionnaire for the assessment of ERS in a community-screened sample of children and adolescents aged 8 to 12 years. The results of an exploratory principal axis analysis suggest a four-factor structure, comprising one factor of *Dysfunctional ERS* and the three functional factors *Distraction*, *Problem-Solving*, and *Social Support*. (Dys-)functionality of the factors was supported by correlations with measures of externalizing and internalizing psychopathology. This structure yielded an adequate model fit in the following CFA. The four-factor structure of the FRUST was invariant across different age groups and gender. However, the analyses did not support scalar invariance across AD/NoAD children, suggesting that the FRUST is not ideal to discriminate between these groups.

Similar to the findings of Cracco and colleagues [34], our results suggest a more complex structure than would be depicted by the mere differentiation between functional and dysfuntional ERS. Whereas Cracco and colleagues [34] proposed a three- or four-factor structure with one functional and two or three dysfunctional factors (three-factor solution: avoidance, dysfunctional thoughts, and aggression; two-factor solution: avoidance and approach), our final structure of the FRUST comprises three functional factors and only one dysfunctional factor. In the FEEL-KJ, five dysfunctional strategies, seven functional strategies, and three ambiguous strategies were identified. The dominance of the functional factors in our sample might be due to the comparatively larger number of functional items in the FRUST compared to the FEEL-KJ, as the 18 newly conceptualized items were primarily intended to represent functional techniques whose acquisition could be targeted in therapy. Seventeen of these 18 items grouped accordingly with other functional items. Similarly, the six items from the three ambiguously (mal-)adaptive strategies in the FEEL-KJ mostly grouped with items of one of the functional ER factors. Thus, it comes as no surprise that the one dysfunctional factor could not be differentiated further. However, the differentiation of the functional factors is interesting, and it remains to be investigated in future studies whether this more detailed assessment of functional ERS has additional predictive value and/or use in therapeutic settings.

In the course of the analysis, two items were excluded due to low factor loadings on all factors, and six additional items were excluded due to similarly high factor loadings on one of the functional factors and the dysfunctional factor. This seemingly puzzling finding may be explained by the specific symptomatology of each individual child. For instance, item 8 ( “*… think that this is my own problem”*) or item 10 ( *“… keep thinking about why I feel bad without finding a solution”*) can be an functional strategy for a child with externalizing symptomatology, who might benefit from looking at their own misbehavior before acting out. By contrast, it might be a dysfunctional strategy for a child with depression, who already focuses on his own behavior in a dysfunctional way. Item 10 might also entail the aspect of “thinking” on the functional side and of “not finding a solution” on the dysfunctional side. Items 31, 33, and 34 were originally thought of as functional items, but include a wording (“*blow off steam*”, “*hit*”, “*make a fist*”) that can evoke associations with aggressive behavior in some children and may therefore potentially explain the cross-loadings on the functional and dysfunctional factors. Item 48 ( *“… do something on my computer/smartphone/tablet”*) can be a functional regulation of a negative emotion, but when used excessively it might be a dysfunctional way to regulate one’s emotions that even has addictive potential. Interestingly, we found that item 16 ( *“… try to find the mistake in my own behavior”*) grouped with items representing problem-solving strategies, suggesting that it might be an functional strategy in the present sample, though it was originally found to represent a maladaptive ERS in the FEEL-KJ. The different assignment of this item might be due to the fact that children with externalizing symptomatology were overrepresented in our sample, suggesting that the strategy of finding mistakes in their own behavior might be rather functional in these children. This points at the important issue of whether a strategy should be classified as functional or dysfunctional. It has been suggested that the functionality of a certain strategy depends on the individual’s symptom background [67], the combination with other strategies (e.g. distraction has been found to be functional when combined with acceptance strategies and dysfunctional when combined with avoidance strategies [68];, the extent and flexibility of use of a strategy (e.g. more expression regulation might lead to overregulation and inhibition [69];), as well as the context [4]. It is important to keep this in mind when using questionnaires to assess ERS profiles, as the functionality of a strategy depends on a number of aspects that need to be considered for each individual case. If, for example, a child scores very high on the factor *Distraction* but low on all other factors, this may indicate a lack of flexibility and an extensive use of this strategy, which might even be dysfunctional for this particular child.

With regard to results on the group level, we found that the positive correlations between the functional scales as well as the negative correlations between the functional scales and the dysfunctional scale supported the construct validity of the FRUST. In terms of external correlates, there was a clear pattern of negative correlations of the primarily functional scales *Distraction*, *Problem-Solving*, and *Social Support* with AD, ADHD, ODD^D^, and the three assessed scales of the CBCL (*Anxious/Depressed*, *Attention Problems*, and *Aggressive Behavior*). Moreover, positive correlations emerged between the primarily dysfunctional scale and AD, ADHD, ODD^D^ and the three assessed scales of the CBCL. These correlations between the ER scales and measures of psychopathology support the classification of the four factors as generally either functional or dysfunctional in the present sample. As emotion dysregulation is assumed to be strongly related to AD [16–18], the positive correlation of AD with the dysfunctional scale and the negative correlation of AD with the functional scales support convergent validity. Given that we assessed externalizing (ADHD, ODD^D^, CBCL *Aggressive Behavior* scales), transdiagnostic (AD, CBCL *Attention Problems scale*), and internalizing (CBCL – *Anxious/Depressed* scale) correlates, the observed correlations with ER can thus be interpreted as an indication of construct validity.

In the present analyses, we found no significant correlations between any of the FRUST scales and age, and only a small correlation of the two functional scales *Distraction* and *Social Support* with gender, suggesting that girls use these strategies more often than boys do. With regard to social support seeking, the findings are in line with previous studies demonstrating a higher use of the strategy *Social Support* in girls than in boys [70]. The frequent finding that girls generally show a more dysfunctional ER [13] could not be replicated in our sample. This may be explained at least in part by the young age of the children in our sample (8–12 years), as some previous studies found that gender effects only emerged later in adolescence [36]. Furthermore, the small age range in the present sample might also explain the lack of associations found between age and ERS. As we examined a restricted and not a representative community sample (children were selected based on their AD symptomatology and categorized into a noAD/AD group), the results have to be interpreted with caution, since potential gender and/or age effects might have been masked by an overrepresentation of a certain group [71]. Longitudinal studies observing the use of ERS during childhood and adolescence in more representative samples are needed in order to better understand the impact of age and gender on ER.

### Strengths and limitations

This study comes with a number of strengths and limitations. In terms of strengths, we developed a shortened, more time-efficient and likely less repetitive version of the FEEL-KJ [33] by assessing the regulation of “unpleasant emotions” in general instead of considering differential responses for dealing with anger, anxiety, and sadness. Moreover, we demonstrated the reliability and validity of this instrument. The present study is the first to validate a shortened version of the self-report FEEL-KJ, as only the parent short version has previously been validated [72]. With the newly conceptualized items in the FRUST, which refer to specific ERS, it is possible to quantitatively capture the therapeutic success regarding the mastery of these specific ERS by administering the questionnaire at different stages of the therapeutic process. The inclusion and description of very specific behaviors might also be advantageous for younger children, as they are less abstract and thus easier to comprehend. Another strength of our study is that – in contrast to other studies on the FEEL-KJ – we assessed the MI of the FRUST factor structure, suggesting an overall stable factor structure of the FRUST.

Nevertheless, several limitations of the study need to be mentioned. First, the cross-sectional design does not allow for any predictive assumptions about the development of ER or any causal interpretations. The few existing longitudinal studies on ER strategies suggest smaller correlations between ER and later psychopathology than found in cross-sectional studies [2]. Second, the FRUST assesses ERS retrospectively, which limits the ecological validity of the ratings. The FRUST does attempt to reduce this problem through the addition of items assessing very specific strategies (it is potentially easier to recall counting to ten or doing a relaxation exercise in a challenging situation than it is to recall trying “… to make the best of a situation”). However, the problem of lacking ecological validity still remains. A combination of the FRUST with other methods such as ecological momentary assessment [9, 14], in which participants are asked to rate their current feelings and thoughts over a longer time period in a naturalistic setting, might be valuable to obtain a more accurate picture of ERS. Third, the FRUST conceptualizes ERS independently of the context, which is opposed to Thompson’s [4] understanding of ER as being strongly dependent on the current situation. Previous findings suggest that the degree of adaptiveness of functional strategies tends to depend on the situation (e.g. problem solving can only be functional if there is an actual problem to solve), whereas dysfunctional strategies like rumination are indeed dysfunctional in most cases [3]. Fourth, the combination of the regulation of sadness, anger, and anxiety into “unpleasant emotions” can be criticized, as there is evidence to suggest that different strategies are used depending on the emotion to be regulated. For instance, social support and avoidance have been shown to be used more frequently for the regulation of sadness, whereas the strategies suppression and rumination are employed more often when attempting to regulate anxiety or anger [11]. Moreover, it is possible that children or adolescents interpret the introductory phrase “When I feel bad” in different ways, or that individual children interpret it differently depending on the specific emotion. On the other hand, it might also be that this more global description of negative emotions is more appropriate for children in this age range, who might have difficulty in clearly distinguishing between a range of negative emotions. Depending on the research or clinical goal, a decision should be made as to whether a time-efficient approach or a more detailed measure of ERS in the specific case is more useful. Fifth, the FRUST only assesses the regulation of negative emotions and not of positive emotions. Though this is common in instruments measuring ER, future research should additionally focus on the use of functional and dysfunctional strategies in the regulation of positive emotions. These strategies might also constitute an important starting point for therapeutic interventions or, in the case of functional strategies, an important resource. Sixth, as we were not able to confirm scalar invariance of the model with four correlated factors across children with different AD levels, the FRUST in its current form cannot be used to differentiate between these groups. Seventh, as a result of the limited sample size and the large number of items of the FRUST, we decided against splitting the sample in half and first performing an EFA in one half in order to then cross-validate the observed structure in the other half. This approach would have been methodologically stronger and as we did not apply it in this study, future work will have to cross-validate the observed structure to gain information regarding its stability.

In addition to the aforementioned limitations regarding the general construction and validation of the FRUST, there are limitations regarding the (non-)inclusion of certain sociodemographic variables. First, we only considered correlations of ERS with age and gender. The correlations with age are also limited, as we only included children between the ages of 8–12 in our sample and have not assessed the structure of the FRUST in a sample of adolescents. Future research should take further sociodemographic variables as well as age groups into account. For example, Greuel and colleagues [72] found that an immigration background and a lower social status of the parents were related to a higher use of functional strategies in their children, whereas no associations with the use of dysfunctional strategies emerged. Second, as we solely considered German-speaking children, the factor structure, psychometric properties, and the age- and gender-related results cannot be generalized to other countries and cultures. This is particularly problematic as there is evidence that ERS might differ between individualistic and collectivistic cultures in terms of the expression or suppression of emotions [73]. Future research should therefore validate the FRUST in other languages, cultures and age-groups (e.g. adolescents).

## Conclusion

The FRUST, which was developed to assess ERS in a more time-efficient and less repetitive manner than the FEEL-KJ [33], demonstrates a stable, well-interpretable factor structure consisting of three functional factors and one dysfunctional factor, which was strongly invariant to age and gender and weakly invariant to AD level. Moreover, the measure showed good psychometric properties in terms of internal consistency and validity. The inclusion of very specific ERS allows for a continuous monitoring of the therapeutic process and might make the questionnaire more accessible for younger children. We believe that due to these features, the FRUST is a valuable contribution to the assessment of ERS for diagnostic, therapeutic, and scientific purposes.

## Supplementary Information

**Additional file 1: Table S1.** Standardized Factor Loadings (Standard Error) for the Confirmatory Factor Analyses of the Correlated Factors Models with Four Factors.

## Data Availability

The dataset and the code used during the current study are available from the corresponding author upon reasonable request.
