# Supplementary material for: Validation of a new emotion regulation self-report questionnaire for children
Source: BMC Psychiatry. 2022 Dec 22;22:820. doi: 10.1186/s12888-022-04440-x (PMC9773459; doi:10.1186/s12888-022-04440-x)
Supplement: Supplementary file 1 — Additional file 1: Table S1. Standardized Factor Loadings (Standard Error) for the Confirmatory Factor Analyses of the Correlated Factors Models with Four Factors. [file 12888_2022_4440_MOESM1_ESM.pdf]

Supplement

Supporting Information for *Validation of a New Emotion Regulation Self-Report Questionnaire for Children*

BMC Psychiatry

Table S1

Standardized Factor Loadings (Standard Error) for the Confirmatory Factor Analyses of the Correlated Factors Models with Four Factors

Item Number	Item Description<br>*When I feel bad, I…*	Factor loading (SE)
	Functional – Distraction	
3	…think about things that make me happy	.86* (.03)
4	…do something fun	.74* (.03)
17	… remember happy things	.82* (.02)
18	…try to make the best of the situation myself	.88* (.02)
27	…do something I enjoy	.88* (.02)
32	…do a relaxation exercise (e.g. imagine blowing up a balloon in my stomach)	.58* (.04)
35	…count to ten	.38* (.06)
36	…go to a nice place in my mind where I can relax	.68* (.03)
37	…listen to music/a story/watch a movie	.45* (.04)
39	…play/paint/do handicrafts	.58* (.04)
40	…solve riddles/read	.55* (.04)
46	…eat something tasty	.44* (.04)
47	…lie on my bed and dream about something nice/try to sleep	.45* (.04)
	Functional – Problem-Solving	
1	…try to change what made me feel bad	.52* (.04)
6	…try to make the best of it	.73* (.03)
11	…think about what I could do	.81* (.02)
12	…tell myself that the problem is not that bad	.59* (.04)
15	…try to forget what put me in a bad mood	.63* (.03)
16	…try to find the mistake in my own behavior	.61* (.04)
20	…think that it will pass	.60* (.04)
21	…accept what makes me feel bad	.55* (.04)
22	…show my bad mood without annoying others	.53* (.04)
28	…think about a solution	.86* (.02)
29	…tell myself that it is not that important	.46* (.04)
42	…try to stay cool and see the problem from another perspective	.73* (.03)
43	…try to think smart instead of obsessing about it	.83* (.02)
44	…leave the situation	.78* (.02)
45	…recognize the situation early that made me feel bad	.73* (.03)

	Functional – Social Support	
2	…tell someone about how I feel	.79* (.03)
5	…keep my feelings to myself	.36* (.05)
14	…say that I am in a bad mood	.59* (.04)
19	…go to someone who might be able to help me	.91* (.02)
41	…talk to someone about it	.77* (.02)
	Dysfunctional Strategies	
7	…do not want to see anyone	.64* (.05)
9	…do not feel like doing anything	.61* (.05)
13	…start a fight	.73* (.04)
23	…take my bad mood out on others	.89* (.04)
24	…cannot get it out of my head	.36* (.05)
30	…cannot do anything against my bad mood	.63* (.05)
38	…hurt myself	.56* (.06)

Note. SE = standard error.

* = $p < .001$
